# Obligately aerobic human gut microbe expresses an oxygen resistant tungsten-containing oxidoreductase for detoxifying gut aldehydes

**DOI:** 10.3389/fmicb.2022.965625

**Published:** 2022-08-16

**Authors:** Michael P. Thorgersen, Gerrit J. Schut, Farris L. Poole, Dominik K. Haja, Saisuki Putumbaka, Harriet I. Mycroft, Willem J. de Vries, Michael W. W. Adams

**Affiliations:** Department of Biochemistry and Molecular Biology, University of Georgia, Athens, GA, United States

**Keywords:** human gut microbiome, aerobe, aldehyde oxidation, benzaldehyde, tungsten

## Abstract

*Brevibacillus massiliensis* strain phR is an obligately aerobic microbe that was isolated from human feces. Here, we show that it readily takes up tungsten (W), a metal previously associated only with anaerobes. The W is incorporated into an oxidoreductase enzyme (BmWOR) that was purified from native biomass. BmWOR consists of a single 65 kDa subunit and contains a single W-pyranopterin cofactor and a single [4Fe-4S] cluster. It exhibited high aldehyde-oxidizing activity with very high affinities (apparent K_m_ < 6 μM) for aldehydes common in the human gut and in cooked foods, including furfural, propionaldehyde, benzaldehyde and tolualdehyde, suggesting that BmWOR plays a key role in their detoxification. *B. massiliensis* converted added furfural to furoic acid when grown in the presence of W, but not in the presence of the analogous element molybdenum. *B. massiliensis* ferredoxin (BmFd) served as the electron acceptor (apparent K_m_ < 5 μM) for BmWOR suggesting it is the physiological electron carrier. Genome analysis revealed a Fd-dependent rather than NADH-dependent Complex I, suggesting that WOR not only serves a detoxification role but its aldehyde substrates could also serve as a source of energy. BmWOR is the first tungstoenzyme and the first member of the WOR family to be obtained from a strictly aerobic microorganism. Remarkably, BmWOR oxidized furfural in the presence of air (21% O_2_, v/v) but only if BmFd was also present. BmWOR is the first characterized member of the Clade 83 WORs, which are predominantly found in extremely halophilic and aerobic archaea (Clade 83A), with many isolated from food sources, while the remaining bacterial members (Clade 83B) include both aerobes and anaerobes. The potential advantages for microbes found in foods and involved in human gut health that harbor O_2_-resistant WORs, including in *Bacillus* and *Brevibacillus* based-probiotics, are discussed.

## Introduction

The group 6 elements tungsten (W) and molybdenum (Mo) are cofactors in a variety of enzymes with roles in the global cycles of many elements, including C, N, S, As and Se (Hille et al., 2014). The W or Mo atom is incorporated into their active sites as part of a pyranopterin cofactor. In spite of their chemical similarity, however, Mo is ubiquitous in biology while W is rarely utilized and is generally regarded as a Mo antagonist (Andreesen and Ljungdahl, 1973; White et al., 1989; Hille et al., 2014; Niks and Hille, 2019). There are four phylogenetically distinct families of pyranopterin-containing enzymes. Three of them utilize Mo and these are the DMSO reductase (DMSOR), xanthine oxidase (XO), and sulfite oxidase (SO) families, although there are some key W-utilizing exceptions in the DMSOR family (Hille et al., 2014). The fourth is the tungsten oxidoreductase or WOR family that primarily uses W (Mukund and Adams, 1996). In contrast to the extremely well-studied Mo-enzymes, only a few WOR enzymes have been characterized and they catalyze a low potential redox reaction, the oxidation of various aldehydes to their corresponding acid (Mukund and Adams, 1995; Roy et al., 2001; Weinert et al., 2015; Arndt et al., 2019; Scott et al., 2019). From phylogenetic analysis, the family of WOR enzymes is unexpectedly large and diverse and can be divided into 92 distinct clades, of which only 4 contain a WOR with a known physiological function (Schut et al., 2021). Surprisingly, 24 of the WOR family clades contain representatives in organisms that are part of the Unified Human Gastrointestinal Protein (UHGP) catalog (Almeida et al., 2021) suggesting that W plays a role in the human gut microbiome.

To date all characterized WOR family members have been isolated from strict anaerobes or facultative anaerobes and these enzymes are rapidly inactivated, often within seconds, upon exposure to oxygen. *Pyrococcus furiosus*, a strictly anaerobic hyperthermophilic archaeon, encodes five WOR proteins, three of which have known physiological functions; formaldehyde ferredoxin oxidoreductase (FOR), glyceraldehyde-3-phosphate oxidoreductase (GAPOR) and aldehyde ferredoxin reductase (AOR). These are involved in the primary metabolism of both carbohydrates and amino acids and all have been shown to be extremely oxygen sensitive (Mukund and Adams, 1995; Roy et al., 1999; Roy and Adams, 2002). The most oxygen-resistant WOR-type enzyme reported so far is the NAD^+^-dependent WOR purified from the strictly anaerobic bacterium *Aromatoleum aromaticum,* which has a half-life of approximately 1 h in air (Arndt et al., 2019). The *A. aromaticum* WOR is also very unusual in using NAD^+^ as the physiological electron acceptor as all other WOR family members utilize the [4Fe-4S]-containing redox protein ferredoxin (Fd). Fd rather than NAD(H) is typically the major electron carrier in the central metabolism of anaerobic microorganisms. These include enzymes that oxidize various 2-ketoacids, such as pyruvate and isovalerate Fd oxidoreductases (Gibson et al., 2016) and the WOR family member (Clade 20 and Clade 23) that oxidizes glyceraldehyde-3-phosphate in some Bacteria (Scott et al., 2019) and Archaea (Mukund and Adams, 1995).

It was recently proposed that the W-containing WOR-type enzymes present in some human gut microbes are involved in oxidizing toxic aldehydes that are present in cooked foods and generated as antimicrobials by gut microbial metabolism (Schut et al., 2021). Specifically, two WORs were characterized from a model strictly anaerobic gut microbe and were shown to convert toxic gut aldehydes to the corresponding acid while reducing Fd. One of the two WORs was a so-called electron bifurcating enzyme that simultaneously reduced NAD and Fd and it was hypothesized that such a strategy enabled the removal of extremely low concentrations of aldehydes in the gut environment, even in the presence of high concentrations of the acids (Schut et al., 2021).

Herein we focus on *Brevibacillus massiliensis* strain phR^T^, which was isolated from human feces and was reported to be endospore-forming and an obligate aerobe (Hugon et al., 2013). Its genome consists of a single 5.05 Mbp chromosome with no plasmids encoding 5,051 proteins. Interestingly, these include a member of the WOR family and a [4Fe-4S] cluster Fd. The putative *B. massiliensis* WOR, now termed BmWOR, is a member of the newly classified Clade 83 phylogenetic group, a member of which has yet to be characterized (Schut et al., 2021). Since a WOR enzyme has yet to be purified from a strict aerobe, it was of great interest to determine if *B. massiliensis* could also grow anaerobically and if a W-containing WOR was produced under aerobic growth. No aerobic microorganism has been previously shown to utilize W. Other than this, however, no information is available on *B. massiliensis,* although other *Brevibacillus* and related *Bacillus* species that are also aerobic and endospore-forming are used as human and animal probiotics (Sanders et al., 2003).

## Materials and methods

### Growth of microorganism

*Brevibacillus massiliensis* (DSM 25447) was obtained from the DSMZ-German Collection of Microorganisms and Cell Cultures in Braunschweig, Germany. The base medium for *B. massiliensis* contained 2 g/l yeast extract as a carbon source (unless otherwise stated), 4.7 mM NH_4_Cl, 1.3 mM KCl, 2 mM MgSO_4_, 0.2 mM NaCl, 1.2 mM NaHCO_3_, 5 mM NaH_2_PO_4_, and 0.1 mM CaCl_2_ with 1X trace elements. The trace elements (1,000X) contained 65.4 mM nitrilotriacetic acid, 25.3 mM MnCl_2_^.^4H_2_O, 7.4 mM FeCl_3_^.^6H_2_O, 7.7 mM CoCl_2_^.^6H_2_O, 9.5 mM ZnCl_2_, 0.4 mM CuCl_2_^.^2H_2_O, 0.4 mM AlK(SO_4_)_2_^.^12H_2_O, 1.6 mM H_3_BO_3_, 1.0 mM (NH_4_)_2_MoO_4_^.^4H_2_O, 1.9 mM NiCl_2_^.^6H_2_O, 0.8 mM Na_2_WO_4_^.^2H_2_O, and 1.1 mM Na_2_SeO_3_. The pH of the medium was adjusted with 5 M NaOH to 7.0 and was filter sterilized using a 0.2 μM filter before use. For Mo and W limited medium, Milli-Q filtered water was used to make up the growth medium, and (NH_4_)_2_MoO_4_^.^4H_2_O and Na_2_WO_4_^.^2H_2_O were not added to the trace elements solution. All growths were performed at 35°C and were monitored in a Bioscreen C (Thermo Labsystems, Milford, MA) with low agitation by measuring OD_600_. For anaerobic growth, the Bioscreen C was placed in an anaerobic chamber (Plas Labs, Lansing, MI) under an atmospheric of 95% Ar and 5% H_2_. For growth in 100 ml bottles, 50 ml of sterile base medium was added to the bottles that were either sealed (with a headspace of 20% O_2_ and 80% Ar) for anaerobic growth or covered with autoclave cloth for aerobic growth. Bottles were incubated with shaking at 40 rpm and growth was monitored measuring the OD_650_ using a Molecular Devices Spectra Max 190 UV–Vis spectrophotometer (San Jose, CA). Growth curves were performed in biological triplicate with error bars representing the standard deviation.

To obtain biomass for the purification of BmWOR, the organism was grown in a medium containing 5 g/l yeast extract, 1.2 mM NaHCO_3_, 10 mM MOPS, 1 mM KH_2_PO_4_, 9.3 mM NH_4_Cl, 4.4 mM KCl, 1.6 mM MgCl_2_^.^6H_2_O, and 1.0 mM CaCl_2_^.^2H_2_O, with vitamins and trace elements prepared as described (Widdel and Bak, 1992). The pH of the medium was adjusted with 5 M NaOH to 7.0 and was filter sterilized using a 0.2 μM filter before use. Multiple flasks (2 l) filled with 1 l of medium were incubated at 35°C and shaking at 40 rpm to late log phase before harvesting cells (6,900 × g for 10 min). Cells were frozen and stored at −80°C until use.

### Purification of WOR and ferredoxin

All purification steps were performed anaerobically either in a Coy anaerobic chamber (95% Ar, 5% H_2_) or using sealed bottles and flasks with an Ar headspace containing buffers degassed with Ar under positive pressure with a constant flow of Ar. Frozen cells (50 g wet weight) were thawed and resuspended in 200 ml of buffer (50 mM HEPES pH 7.5, 5% glycerol (v/v), 5% trehalose (w/v), 1 mM cysteine, 0.1 mM PMSF and 50 mg/l DNAse). Cells were broken by sonication on ice (30-s intervals, amplitude 60; Qsonica, model Q55). An extract of soluble proteins was prepared by ultracentrifugation at 100,000 × g for 1 h. Anion exchange chromatography was carried out using a 50-ml QHP custom column (XK 26/20 Cytiva, Marlborough, MA) equilibrated with 25 mM HEPES (pH 7.5), containing 1% trehalose (w/v), and 1 mM cysteine. Bound proteins were eluted with a linear 1 l gradient for 0 to 500 mM NaCl in the same buffer at a flow rate of 8 ml/min. Benzaldehyde oxidation activity eluted as a single peak when ~250 mM NaCl was applied. Hydrophobic interaction chromatography was carried out on a custom 50 ml Phenyl Sepharose HP column (XK 26/20 Cytiva) equilibrated with 25 mM HEPES (pH 7.5), containing 0.5 M (NH_4_)_2_SO_4_, 1% trehalose (w/v), and 1 mM cysteine. Bound proteins were eluted at a flow rate of 7.5 ml/min and a 500 ml linear gradient from 500 to 0 mM (NH_4_)_2_SO_4_ in the same buffer. Benzaldehyde oxidation activity eluted as a broad peak between 400 to 150 mM (NH_4_)_2_SO_4_. Size exclusion chromatography was carried out using a HiLoad Superdex 200 prep grade XK 26/60 column (Cytiva) equilibrated with a running buffer of 25 mM HEPES (pH 7.5) containing 300 mM NaCl and 1% trehalose (w/v) at a flow rate of 2.5 ml/min. A 10-ml monoQ (Cytiva) equilibrated with HEPES, pH 7.5, containing 1% trehalose (w/v) was used as the final purification step. WOR eluted as ∼250 mM NaCl was applied to the column. A total of approximately 2 mg of purified WOR was obtained from 50 g (wet weight) of cells. All fractions were collected in Ar-flushed serum vials sealed with butyl rubber stoppers and were stored at 4°C. *B. massiliensis* Fd was purified from the same batch of cells; it eluted from the first anion exchange column when 300 mM NaCl was applied. In the subsequent size exclusion step, Fd eluted from the HiLoad Superdex 200 XK 26/60 column corresponding to a molecular weight of 10 kDa based on visible absorption (400 nm) and iron analysis.

### Assays

Individual aldehyde oxidation assays were performed in an Agilent Cary 100 UV/Vis spectrometer by following the reduction of benzyl viologen at 578 nm (extinction coefficient of 8.65 mM^−1^·cm^−1^; Choe et al., 2014). The reaction mixture (2.0 ml) contained 50 mM HEPES, pH 7.5, 1 mM benzyl viologen, ∼4 μM sodium dithionite, and the enzyme sample, and the reaction was initiated by the addition of various aldehyde substrates (500 μM) as indicated. In case of the Fd-linked assay, the reaction mixture (400 μl) contained ~50 μM BmFd as the electron acceptor and was followed at 425 nm (extinction coefficient of 13.0 mM^-1.^cm^−1^; Aono et al., 1989). Standard assays were conducted at 35°C. Activity is reported in units where one unit (U) catalyzes the reduction of 1 μmol benzyl viologen/min or 1 μmol Fd/min. Specific activity is calculated based on the concentration of protein measured using the Bradford reagent (Bio-Rad; Bradford, 1976). Aldehyde stock solutions (50 mM) were made in 95% ethanol. The 96-well plate aldehyde oxidation assays were performed under anaerobic conditions as previously described (Schut et al., 2021). Reaction mixtures (200 μl) contained 50 mM HEPES pH 7.5, 250 μM aldehyde substrate, 750 μM benzyl viologen, ~4 μM sodium dithionite and 1–100 μg enzyme. The increase in absorbance at 600 nm compared to control assays in the absence of substrate was measured in a Molecular Devices Spectra Max 190 UV–Vis spectrophotometer (San Jose, CA). Relative activity compared to benzaldehyde oxidation activity is reported. Aldehyde oxidation assays in the presence of oxygen were performed in 8 ml stoppered serum vials containing 1 ml 50 mM HEPES, pH 7.5 and were flushed with Ar/H_2_ (95/5). ~7% oxygen was introduced by injecting 500 μl pure O_2_ using a gastight syringe (Vici, Baton Rouge, LA). The reaction of furfural with purified BmWOR (60 μg) and BmFd (10 μM) was followed by measuring O_2_ consumption in the headspace using a Shimadzu GC-8A GC equipped with a molsieve column (Restek 5A 80/100) at 80°C with Ar as carrier gas and a TCD detector. Furfural decrease and the formation of furoic acids were measured using a GC 7890A (Agilent) fitted with a flame ionization detector (FID) and equipped with a carbowax capillary column (Restek stabilwax, 30 m, 0.32 mM, 0.25 μM).

The activities of pyruvate ferredoxin oxidoreductase (POR) and 2-ketoglutarate ferredoxin oxidoreductase (KGOR) were measured by following the reduction of 1 mM benzyl viologen (BV) at 578 nm using 5 mM pyruvate or 2-ketoglutarate as the substrates (Blamey and Adams, 1993; Mai and Adams, 1994). The assay mixtures also contained 2 mM MgCl_2_, 1 mM thiamine pyrophosphate (TPP) and 0.2 mM CoASH. Pyruvate dehydrogenase and 2-ketoglutarate dehydrogenase activities were measured using the same assay mixture except that the electron acceptor was 150 μM 2,6-dichlorophenolindophenol (DCPIP) at 600 nm (extinction coefficient of 12.5 mM^−1^·cm^−1^; Basner and Antranikian, 2014). All activities are reported in units where one unit catalyzes the oxidation of 1 μmol of substrate/min (Bradford, 1976).

### Quantitative RT-PCR

*Brevibacillus massiliensis* cultures (50 ml) were grown in 100 ml bottles aerobically to early log phase (OD_660_ ~ 0.2). Cultures were chilled on ice for 10 min and cells were then harvested by centrifugation (10 min, 6,900 × g). RNA was extracted from cells using a phenol/chloroform extraction method (Schut et al., 2001). RNA was digested with TURBO DNAse (Ambion) to remove contaminating genomic DNA. The quality of the RNA was evaluated by A_260_/A_280_ and QPCR. cDNA synthesis was performed using 1 μg purified RNA with the Affinity Script QPCR cDNA synthesis kit (Agilent). Quantitative RT-PCR was conducted using Brilliant II SYBR Green QPCR Master Mix (Agilent). Primers were designed to amplify ~150 bp product within the target gene. The gene encoding glyceraldehyde-3-phosphate dehydrogenase (WP_019121966.1) was used as the reference gene.

### Metal determination

Metal concentrations were measured as previously described (Schut et al., 2021). In brief, samples were diluted between 1:20 and 1:100 into 2% (v/v) trace-grade nitric acid (VWR) in acid-washed 15-mL polypropylene tubes, depending on the expected metal and salt concentration. Samples were incubated at a 37°C temperature with shaking for at least 1 h before centrifugation at 3,000 × g for 20 min to pellet debris. Samples were analyzed by an Agilent 7,900 ICP-MS fitted with MicroMist nebulizer, ultra-high matrix introduction (UHMI) spray chamber, platinum (Pt) cones, x-lens, and an Octopole Reaction System collision cell with He mode (Agilent Technologies). Results are reported as the average of three analytical replicates. Samples reported with standard deviations are from three or more biological replicates.

### Phylogenetic tree and classifications

The 197 Clade 83 WOR family member sequences were extracted from a larger previously published tree (Schut et al., 2021). *Pyrococcus furiosus* AOR (WP_011011461.1; Clade 87) was added as an outgroup. The phylogenetic tree was reconstructed by a neighbor-joining model with a resampling size of 1,000 using Geneious Prime 2022.0.2. Information for each characterized microorganism was located and the metadata manually recorded (Supplementary Table S1). Several microorganisms that have unique Clade 83 WOR sequences have identical species names because they are non-cultured, have multiple WORs and/or are poorly named strains. Strains of a given species strains were individually counted. The classification of the Clade 83 microorganisms were based on optimal growth conditions and are: mesophile (14 to 45°C), thermophile (45 to 80°C) and hyperthermophile (>80°C; Amend and Shock, 2001); slight halophile (up to 3% w/v or 0.51 M NaCl), moderate halophile (3–15% w/v or 0.51–2.6 M NaCl) and extreme halophile (>15% w/v or 2.6 M NaCl; Margesin and Schinner, 2001); neutrophiles (pH 5.0–8.0), alkaliphiles (pH > 8.0, Grant et al., 1990). The tree was annotated using our manually curated characterization metadata table and then visualized using iTOL (Letunic and Bork, 2021).

## Results

### WOR Clade 83 is enriched in aerobic and extremely halophilic microorganisms

**Figure 1:**
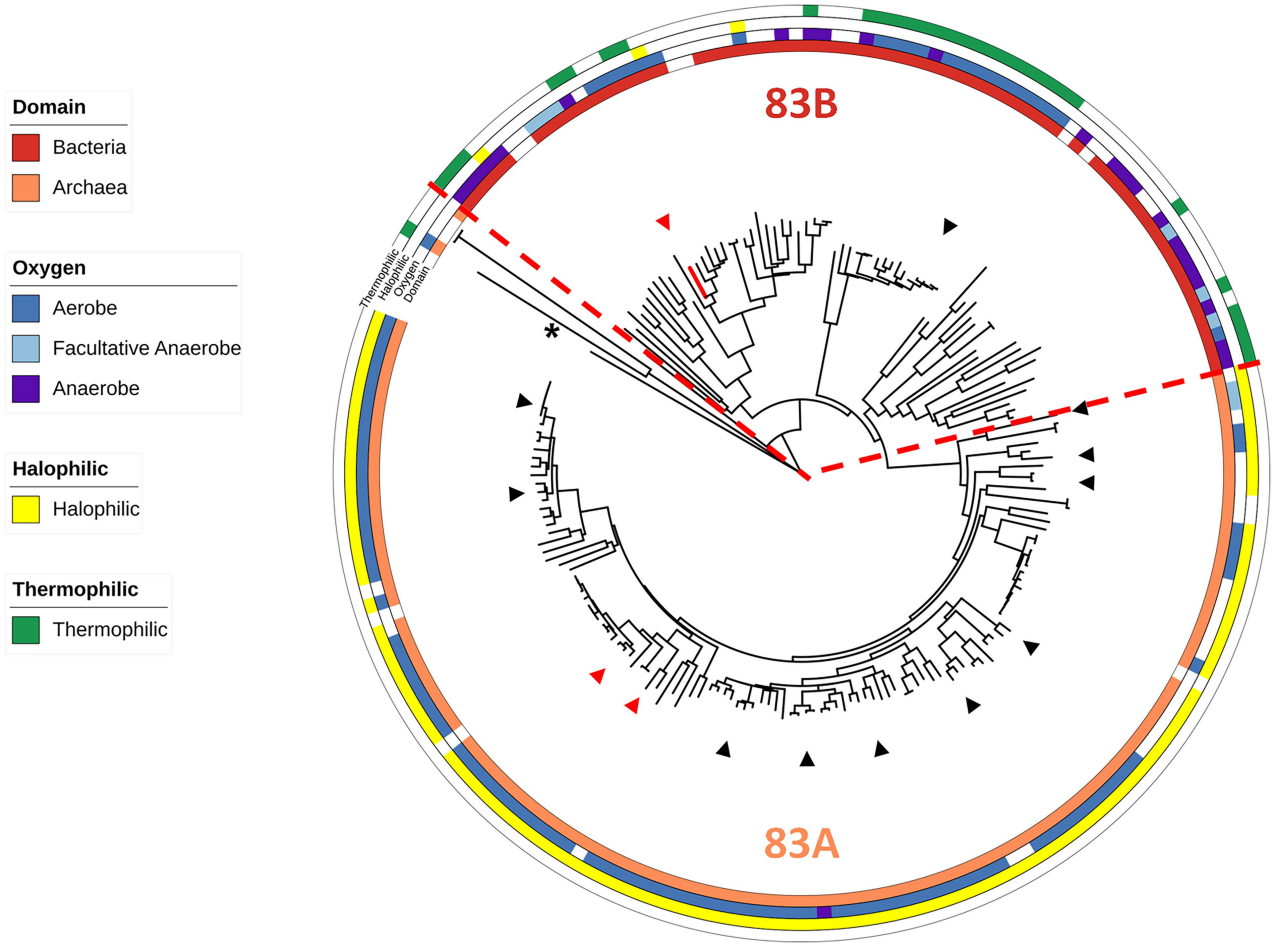
Characteristics of WOR Clade 83 organisms. An expanded and modified view of the previously published Clade 83 member proteins (Schut et al., 2021), now showing the domain of the host organism and their oxygen, temperature and salt requirements (see Supplementary Table S1). The red dotted line marks a point of phylogenetic divergence between the Archaea and Bacteria. *B. massiliensis* WOR is colored red and *P. furiosus* AOR from Clade 87 (Schut et al., 2021) was used as the outgroup (shown by the asterisk). Red triangles represent human gut microbiome-related organisms while black triangles represent food-related organisms.

Of the 197 sequences within the Clade 83 WOR, 124 are from archaeal samples and 73 are from bacterial samples, and these split into two distinct subclades, designated 83A and 83B, respectively (Figure 1; Supplementary Table S1). Of these samples, 119 archaeal species and 52 bacterial species were cultured and had published information available for further classification regarding the effects of temperature, salt and/or pH on growth (Figure 1; Supplementary Table S1). A total of 112 out of 119 cultured archaea were extreme halophiles (optimal growth with more than 15% w/v or 2.6 M NaCl), and seven were moderate halophiles (optimal growth with 3–15% w/v or 0.51–2.6 M NaCl). A total of 95 out of the 119 archaea were obligate aerobes, 3 were anaerobes and 21 were facultative anaerobes, while 110 were mesophiles, eight were thermophiles (T_opt_ > 45°C) and one was a hyperthermophile (T_opt_ > 80°C). Of the 52 cultured bacteria in the clade, 25 were mesophiles and 27 were thermophiles, while 6 were extreme halophiles, 3 were moderate halophiles, 37 were slight halophiles (optimal growth with less than 3% w/v salt or 0.51 M NaCl) and four were halotolerant (not requiring salt for growth but able to grow in the presence of salt). Of the bacteria, 29 were aerobes, 14 were anaerobes, and 9 were facultative anaerobes (Figure 1; Supplementary Table S1). Of the 29 distinct genera of halophilic archaea with a Clade 83 WOR, 3 genera had species that were found to have been isolated from human-related samples (fecal, skin, oral, etc.) and 11 had species that have been isolated from food-related samples. A total of 14 of the 29 genera have species that have been isolated from samples taken from salt-rich environments and salterns (Lee, 2013; Henriet et al., 2014; Gibtan et al., 2017; Seck et al., 2018). Of the 17 distinct bacterial genera, two were found in human-related samples, one in food-related samples and one in a saltern (Kumar et al., 2015; Quigley et al., 2016; Yu et al., 2016; Satari et al., 2021). Hence, the WOR of *B. massiliensis* appears to be prototypical of WORs found in aerobes that are predominantly extremely halophilic Archaea, many found associated with human microbiomes, like *B. massiliensis,* or in foods. A fundamental question was, therefore, is *B. massiliensis* able to grow anaerobically and is it halophilic?

### *B. massiliensis* is an aerobe that utilizes peptides for growth

**Figure 2:**
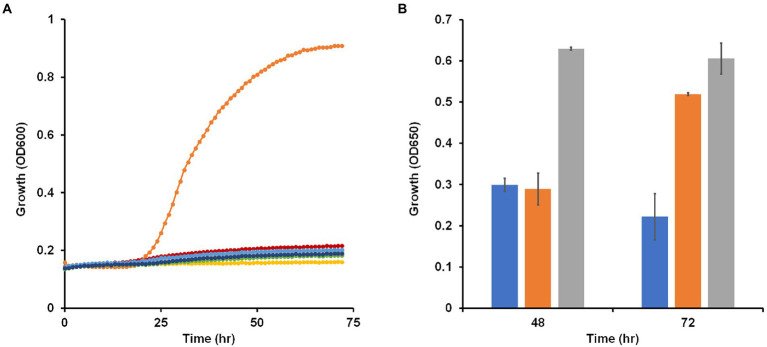
Growth of *Brevibacillus massiliensis* under various oxygen regimes and presence of potential electron acceptors. **(A)** *Brevibacillus massiliensis* was grown on base medium containing 5 g/l yeast extract and 5 g/l glucose aerobically (orange) or anaerobically with different electron acceptors: none (red), 20 mM nitrate (green), 2.5 mM nitrite (blue), 90 mM trimethylamine N-oxide (dark blue), 70 mM dimethyl sulfoxide (gray), or 40 mM fumarate (yellow). **(B)** *Brevibacillus massiliensis* was grown aerobically on base medium containing 2 g/l yeast extract in either sealed (blue and orange) or unsealed (gray) bottles. Growth (OD_650_) was measured at 48 h before some of the sealed tubes were unsealed (orange) and growth (OD_650_) was then measured again after a further 24 h.

Most *Brevibacillus* species are strict aerobes with *Brevibacillus laterosporus*, a facultative anaerobe, being an exception (Shida et al., 1996). *B. massiliensis* was originally isolated aerobically from human feces and previous characterization of the strain as an aerobe was based on the lack of growth under anaerobic or microaerophilic conditions using GENbag anaer and GENbag microaer sachets (Hugon et al., 2013). To further test the status of *B. massiliensis* as a strictly aerobic organism, we analyzed the genome looking for enzymes indicative of an anaerobic respiratory metabolism. No obvious genes encoding nitrate, DMSO or TMAO reductases were found. The genome does encode a fumarate reductase-related enzyme and a transcriptional regulator of the Fnr family and these may or may not be involved in anaerobic metabolism. Since the genome assembly is in 132 contigs, it is possible that genes are missing from the overall sequence of the organism. Therefore, we attempted to grow *B. massiliensis* on base medium containing 5 g/l glucose and 5 g/l yeast extract as carbon sources anaerobically with and without various terminal electron acceptors, including nitrate, nitrite, DMSO, TMAO, and fumarate. None of these conditions resulted in anaerobic growth of the strain while the same media supported robust growth aerobically (Figure 2A).

To further confirm the aerobic requirement of *B. massiliensis* for detectable growth, it was grown aerobically in capped and uncapped bottles on the base medium, which contains 5 g/l yeast extract as the carbon source. Growth in the capped bottles stopped due to lack of O_2_ resulting in a lower OD_600_ at 48 h of 0.3 versus 0.6 for uncapped bottles with fully aerated conditions. When the bottles were uncapped leading to full aeration of the cultures, growth resumed (Figure 2B). The O_2_ concentration in the solution of the capped bottles when growth ceased was 40 ± 1 μM indicating that *B. massiliensis* is unable to grow under oxygenation conditions below this concentration (air-saturated water contains approximately 200 μM O_2_ at 35°C). In stationary phase, O_2_ utilization continued, and the culture consumed the remaining O_2_ within 24 h. When the organism was grown aerobically on base medium containing only 0.5 g/l yeast extract, growth reached an OD_600_ of about 0.51 in after a 60 h period, compared to about 0.9 when 5 g/l yeast extract was used. The former condition was used to determine if other carbon sources could be utilized. As shown in Supplementary Figure S1, a small increase in growth was observed with the addition of pyruvate (OD_600_ 0.63), but no growth increase was observed with glucose, fructose, galactose, mannitol, sorbitol, xylitol, gluconate, lactate, L-arabinose, sucrose, trehalose, cellobiose, ethanol, fumarate or glycerol. In contrast, addition of casein (2 g/l) greatly stimulated the growth of the organism (OD_600_ 0.77; Supplementary Figure S1). We conclude that *B. massiliensis* readily utilizes amino acids present in yeast extract and casein as carbon sources but is unable to degrade other carbon sources including various carbohydrates.

### W-containing proteins of *B. massiliensis*

The *B. massiliensis* genome has 5,051,018 bp in a single chromosome with no plasmids in 132 contigs (Hugon et al., 2013). We analyzed the *B. massiliensis* genome for known W- and Mo-associated genes. The organism contained both the *tupABC* and *modABC* genes that encode tungstate (WO_4_^2−^) and molybdate (MoO_4_^2−^) transport systems, respectively. In addition to the Clade 83 WOR, the genome contained genes encoding six other pyranopterin enzymes, five of the Mo-containing XO family and one of the (almost exclusively) Mo-containing DMSO family. However, none of these Mo-related genes were sufficiently similar to characterized genes to assign a function. Indeed, nitrate, DMSO and TMAO reductases are all members of the DMSO family but clearly none are encoded by the DMSO family member gene identified in the genome. The previously characterized WORs from a model (anaerobic) gut microbe were encoded by two- or five-gene operons with the latter encoding an electron bifurcating enzyme (Schut et al., 2021). In contrast, the WOR of *B. massiliensis* is encoded by a single gene (WP_019122299.1) and that is on the opposite strand of the surrounding genes and is next to a transcriptional regulator that is adjacent to the *tupABC* tungstate transport genes (Supplementary Figure S2), suggesting regulation by tungstate. The *B. massiliensis* genome also encodes a single [4Fe-4S]-cluster Fd (WP_019122927), which could be the *in vivo* electron acceptor for the putative WOR enzyme.

### Effects of W on *Brevibacillus massiliensis*

To investigate if *B. massiliensis* was able to assimilate W or Mo from the growth medium and if this had any effect on its growth, a medium containing minimal W and Mo was developed using high purity chemicals and Milli-Q distilled water. Analysis by ICP-MS showed that this contained 2 nM Mo and < 0.7 nM W, which were present as contaminates from other medium components. In the following experiments, the concentrations of W (added as tungstate, 100 nM WO_4_^2−^) and/or Mo (added as molybdate, 100 nM MoO_4_^2−^) refer to that intentionally added in addition to the contaminating amounts (Supplementary Figure S3 A). The growth of *B. massiliensis* with or without added W and/or Mo showed no obvious differences (Supplementary Figure S3 B). We then isolated RNA from cultures grown using the same four different Mo/W conditions and used qPCR to determine gene expression changes in response to the presence of W and/or Mo. While expression of *tupA* and *modA* were significantly decreased by the addition of W or Mo to the medium, respectively, the expression of the WOR gene and of the DMSOR-like gene were unaffected by W and/or Mo addition (Supplementary Figure S3 C).

**Figure 3:**
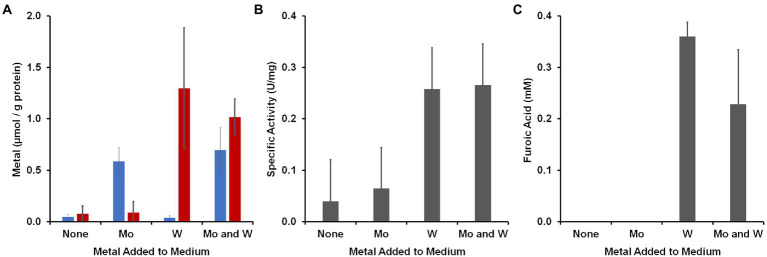
Accumulation of metals by *B. massiliensis* and their effects on benzaldehyde oxidation activities. The organism was grown in base medium containing 2 g/l yeast extract with and without added W or Mo (100 nm). **(A)** Intracellular accumulation in the cytoplasmic extract of W (red) and Mo (blue). **(B)** Benzaldehyde oxidation activity (benzyl viologen as an electron acceptor) in cytoplasmic extracts. **(C)** Accumulation of furoic acid when the growth medium was supplemented with 1 mM furfural.

To determine to what extent the organism accumulated W and Mo, cytoplasmic extracts were prepared from cells grown with the four different Mo/W media conditions and Mo and W was measured by ICP-MS (Figure 3A). In the absence of added W or Mo, less than 0.1 μmol/g protein accumulated in the cytosolic extract. The W and Mo contents increased approximately 16- and 12-fold, respectively, when either was added and neither metal seemed to significantly alter the uptake of the other when they were both added. However, the aldehyde oxidation activity in the cytoplasm was influenced by added W but not Mo. Benzaldehyde oxidation activity (dye-linked) was about four-fold higher in cytoplasmic extracts that were made from cells grown with 100 nM W compared with those grown without added W regardless of added Mo (Figure 3B). The cytosolic extract prepared with added W and Mo was used in a plate assay in which 48 different aldehydes were screened as possible substrates (Schut et al., 2021). Many of these aldehydes were measured as part of the human gut metabolome (Wishart et al., 2018) while others are found in cooked foods (O’Brien and Morrissey, 1989). As shown in Supplementary Table S2, the highest activities were observed with aromatic compounds found in the gut, such as benzaldehyde, or in foods, such as furfural.

To determine if there was a metabolic phenotype associated with W and physiologically related aldehydes, *B. massiliensis* was grown with and without W in the presence and absence of benzaldehyde or furfural (each 1 mM). Spent media were analyzed for the aldehydes and potential oxidation products, benzoic acid and furoic acid, respectively, at the end of exponential growth (60 h). In the case of both benzaldehyde and furfural addition, no benzaldehyde or furfural (<25 μM) was detected whether W was added or not, indicating the complete catabolism of these aldehydes had occurred. However, in the case of furfural addition, ~ 0.3 mM furoic acid was detected at the end of exponential growth, but only when W was also added to the medium (Figure 3C). With benzaldehyde addition, no benzoic acid was detected whether W was added to the medium or not. These results show that the amount of W in the growth environment of *B. massiliensis* has several impacts on the organism including its intracellular accumulation of W, its aldehyde oxidation activity, and the end products that are generated. We assumed that the aldehyde oxidation activities were associated with the putative W-containing WOR in *B. massiliensis* and thus, benzaldehyde was chosen as the substrate for purification purposes, as will now be described.

### BmWOR contains W and oxidizes multiple gut-related aldehydes

**Figure 4:**
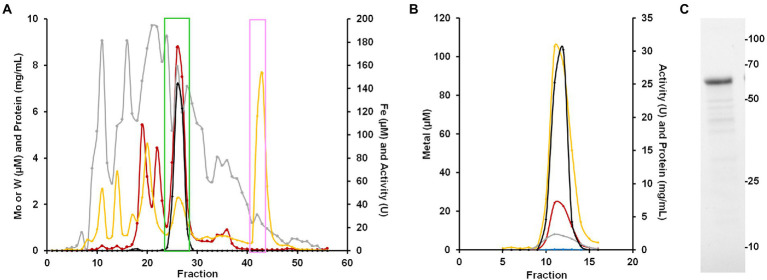
Purification of BmWOR and BmFd. **(A)** Elution profile from the first QHP column showing benzaldehyde oxidation activity (black), protein (gray), Fe (yellow) and W (red). The green box indicates fractions that were pooled for the BmWOR purification and the pink box indicates the fractions that were pooled for the BmFd purification. **(B)** BmWOR elution profile from the final MonoQ column showing benzaldehyde oxidation activity (black), protein (gray), Fe (yellow), W (red) and Mo (blue). **(C)** SDS-PAGE gel of purified BmWOR from the peak MonoQ fraction showing a major band at 65 kDa.

To purify the WOR from *B. massiliensis,* a cytosolic extract was made from 50 g of aerobically grown *B. massiliensis* in a medium to which 100 nm WO_4_^2−^ and MoO_4_^2−^ were added. This was fractionated on a QHP anion exchange column under anaerobic conditions (as previously characterized WOR enzymes from anaerobes are extremely oxygen sensitive) and the resulting fractions were analyzed for their protein concentration, benzaldehyde oxidation activity and metal content (by ICP-MS). As shown in Figure 4A, the enzyme(s) responsible for benzaldehyde oxidation activity eluted as a single peak that overlapped a large peak of W and Fe. Two smaller W peaks were also observed eluting from the QHP column but subsequent analysis of these W-containing fractions indicated that the W was not tightly bound to specific proteins and is easily disassociated, and these are currently under study. Besides W and Fe, peaks of several other metals eluted from the QHP column indicating a range of other metalloproteins, including those containing Mo, Mn, Co, Ni, Cu, and Zn (Supplementary Figure S4). The peaks of Co, Mn and Zn were at concentrations above that of W. The Mo peaks were about an order of magnitude lower in concentration than those of W, emphasizing that W-containing proteins appear to play a more significant role in the metabolism of *B. massiliensis.*

The fractions containing the benzaldehyde oxidation activity were further fractionated on a Phenyl Sepharose column, followed by size exclusion (SX200) and a second anion exchange column (MonoQ). The single peak of activity that eluted from the final step coincided with single peaks of W, Fe and protein (Figure 4B). SDS-PAGE gel analysis revealed a single protein band with an apparent mass of approximately 65 k Da (Figure 4C), which corresponds to a monomeric quaternary structure based on the sizing estimate from the SX200 column. MS/MS analysis of the purified enzyme identified the W-containing protein as what will be referred to as BmWOR encoded by gene WP_019122299.1, corresponding to a calculated molecular weight of 65 kDa. The purification table for BmWOR following benzaldehyde oxidation to benzyl viologen activity is shown in Table 1. The enzyme was purified 75-fold with a final specific activity of 7.1 U/mg. From the amino acid sequence, BmWOR is predicted to contain a single tungsto-pyranopterin site and a single [4Fe-4S] cluster. Consistent with this, the protein to metal ratios of the purified enzyme were 0.7 W atoms per protein (expected 1), and 4.3 Fe atoms per W atom (expected 4). The W to Mo ratio in the final enzyme preparation was 141 to 1, indicating that W is strongly favored over Mo as the metal in the pterin center of BmWOR.

**Table 1:** Purification of BmWOR.

Step	Specific Activity (U/mg)	Activity^a^(U)	Protein(mg)	Yield(%)	Fold Purification
		
Cytoplasmic extract	0.09	297	3,115	100	1
QHP	0.47	334	708	112	5
Phenyl Sepharose	1.9	282	145	94	22
SX200	4.8	47	28	16	54
MonoQ	7.1	10.1	4.2	3.4	79

a Activity was measured by benzaldehyde oxidation coupled to benzyl viologen reduction.

The large Fe peak eluting near the end of the gradient applied to the first QHP ion-exchange column was predicted to be Fd, and this was purified by subsequent size exclusion chromatography. The predicted size of the protein encoded by gene WP_019122927 was 7,465 Da. The sequence of BmFd contained four Cys residues in the arrangement predicted to coordinate a single [4Fe-4S] cluster. The spectra of the oxidized and Ti(III) citrate-reduced forms of the protein are shown in Supplementary Figure S5. The Fd was predicted to be the native electron acceptor for the WOR enzyme. This appears to be the case as the enzyme had 10-fold higher specific activity (75.5 U/mg vs. 7.1 U/mg) when the purified Fd was used as the electron acceptor instead of benzyl viologen for the oxidation of benzaldehyde. In this benzaldehyde oxidation assay, the apparent K_m_ for Fd was estimated to be <5 μM.

The aldehyde oxidation plate assay used for the cytoplasmic extract was also used to analyze the purified WOR enzyme (Supplementary Table S2). Very similar results were obtained with both sample types, indicating that WOR alone is most likely responsible for the aldehyde oxidation activities inside the cell. For BmWOR, the highest activities were observed for aromatic aldehydes, including tolualdehyde, benzaldehyde, cinnamaldehyde (trans), 3,4-dihydroxy-benzaldehyde, 2-hydroxylbenzaldehyde, furfural and 5-hydroxymethylfurfural, which are known to be present in the human gut or in cooked foods (O’Brien and Morrissey, 1989; Wishart et al., 2018). High activities were also observed with other gut-related aldehydes including propionaldehyde, glycoaldehyde and 2-methyl-2-butenal. In cuvette-based assays, benzaldehyde had the highest specific activity followed by cinnamaldehyde (trans) and tolualdehyde (Table 2). The apparent K_m_ values for seven tested gut-related aldehydes were all <6 μM, indicating a high affinity of BmWOR for these substrates. When *B. massiliensis* was grown in the presence of tolualdehyde, isovaleraldehyde or phenylacetaldehyde (each 1 mM), no differences in growth were observed. Analysis of RNA isolated from cells grown in the presence of these aldehydes and also benzaldehyde and furfural revealed no significant changes in the expression level of BmWOR (Supplementary Figure S6).

**Table 2:** Aldehyde oxidation activities of BmWOR.

Substrate	Specific Activity^a^	K_m_
	(U mg^−1^)	(μM)
Benzaldehyde	7.1 ± 0.2	4.2 ± 1.0
Cinnamaldehyde (trans)	5.3 ± 0.4	5.6 ± 1.2
Tolualdehyde	4.8 ± 0.9	6.1 ± 2.1
Phenylacetaldehyde	4.2 ± 1.2	5.3 ± 1.6
Furfural (2-furaldehyde)	3.5 ± 0.3	2.1 ± 2.0
Isovaleraldehyde	2.8 ± 0.2	3.6 ± 0.9
Propionaldehyde	1.8 ± 0.1	4.4 ± 1.8

a Aldehyde oxidation activities were determined using benzyl viologen as the acceptor.

### BmWOR is catalytically active in the presence of O_2_

As described above, BmWOR falls into Clade 83 of the WOR phylogenetic tree (Schut et al., 2021). Interestingly, 97% (116) of the Clade 83 Archaea are aerobic (including facultative anaerobes), and of those 93% (109) are extremely halophilic (growing optimally in >15% w/v or 2.6 M NaCl), and of those about 6% (6) of them are thermophiles (45 to 80°C). Similarly, 73% of the Clade 83 Bacteria are aerobic (including facultative anaerobes) but in contrast only one of those is an extremely halophilic (growing optimally in >15% w/v or 2.6 M NaCl) mesophile. In the extremely halophilic Archaea, the intracellular counterion is potassium (da Costa et al., 1998) and it would be of interest to determine if the *B. massiliensis* enzyme was halophilic. However, neither it nor the unrelated WOR (Clade 87) from *Acetomicrobium mobile* (Schut et al., 2021), were significantly affected by high KCl concentrations, with both having ~60% activity when assayed in the presence of 4.0 M KCl and retaining greater than 80% activity after 24 h in 3.0 M KCl. This is in contrast with *B. massiliensis* itself, as it was unable to grow at NaCl concentrations >200 mM. In contrast, while *B. massiliensis* is a mesophile with an optimum growth temperature of 35°C (Hugon et al., 2013), purified BmWOR shows remarkable thermostability, retaining greater than 20% activity for 4 days when incubated at 55°C. For comparison, the Clade 87 WOR from *A. mobile* (Schut et al., 2021), which has an optimum growth temperature of 55°C, lost 50% activity after 12 h at 55°C and was completely inactive after 20 h.

**Figure 5:**
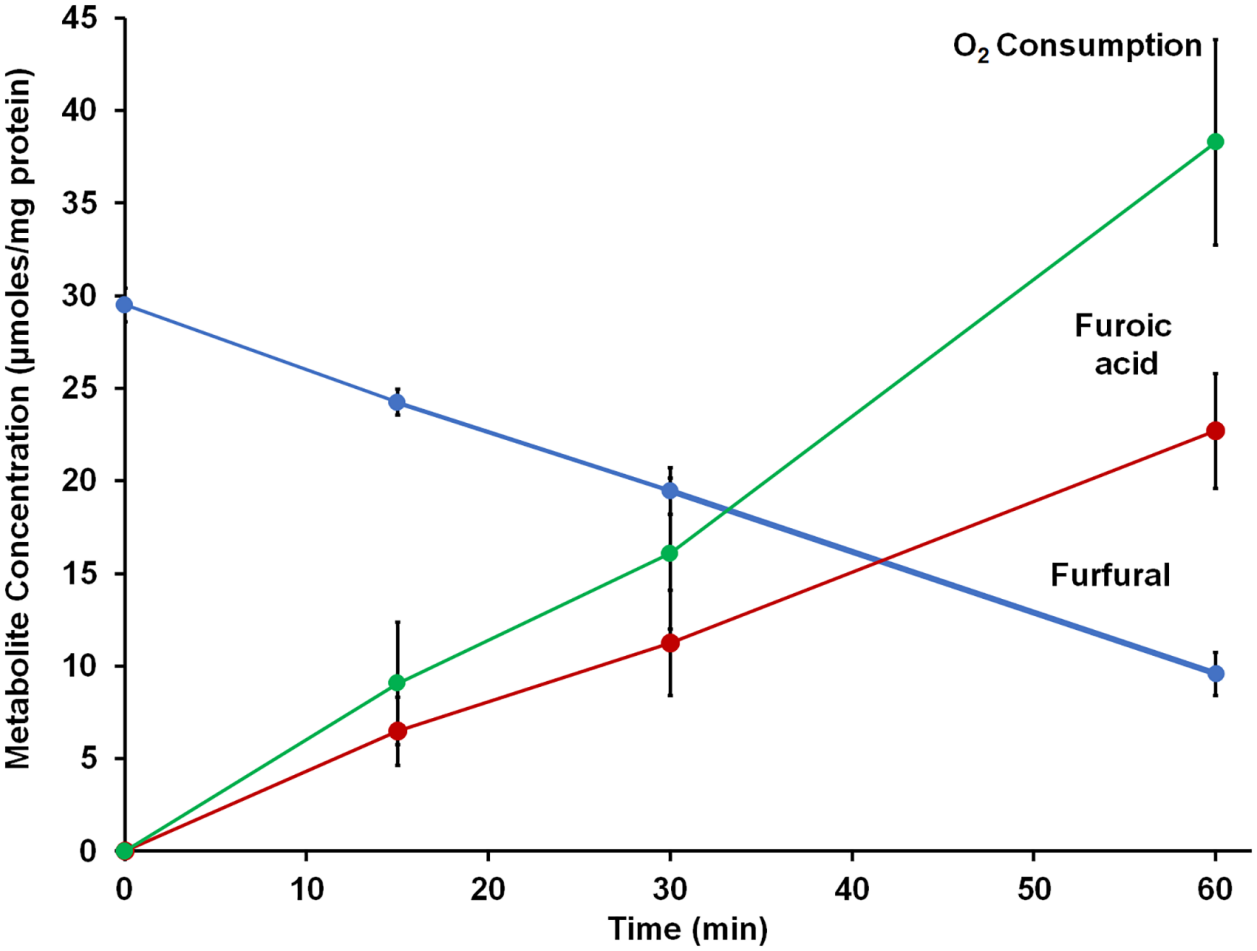
Furfural oxidation activity of BmWOR in the presence of oxygen. Purified BmWOR was assayed using 1.5 mM furfural in the presence of 10 μM *B. massilliensis* Fd in closed serum vials containing 7% (v/) oxygen in the headspace. At indicated intervals, headspace samples were removed to determine the oxygen consumption and liquid samples were removed to determine furfural and furoic acid concentrations.

Since *B. massiliensis* is also an obligate aerobe and BmWOR is the first enzyme of the WOR family to be purified from such an organism, it was of interest to determine both the stability of BmWOR in the presence of O_2_ and also whether it was catalytically active under such conditions. The enzyme was highly resistant to oxygen, retaining 50% activity after 48 h in air-saturated buffer (21% O_2_, ~ 200 μM O_2_), while the *A. mobile* enzyme was completely inactive after only 10 min. To determine if BmWOR is catalytically active in the presence of oxygen, the reaction was carried out in its presence. Oxygen was added (final headspace concentration of 7%, v/v, in Ar) to a closed serum vial containing BmWOR and BmFd (10 μM). The reaction was initiated by the addition of 1.5 mM furfural. BmWOR was indeed active in the presence of oxygen as the reaction could be followed by measuring oxygen consumption in the headspace and of furfural in the liquid phase while close to stoichiometric amounts of furoic acid were produced (Figure 5). BmWOR had similar furfural oxidation activity in air (21% O_2_, ~ 200 μM) but no activity was observed if BmFd was omitted from either reaction. This shows that the enzyme cannot use O_2_ as an electron acceptor directly, rather, O_2_ reacts with and oxidizes the reduced Fd that is produced by the enzyme. The ratio of furoic acid produced to oxygen utilized is approximately 1:2 (Figure 5), indicating that superoxide (O_2_ reduced by one electron) rather than hydrogen peroxide (O_2_ reduced by two electrons) is the initial product of the reaction of oxygen reacting with reduced Fd.

### *Brevibacillus massiliensis* ferredoxin-dependent metabolism

**Figure 6:**
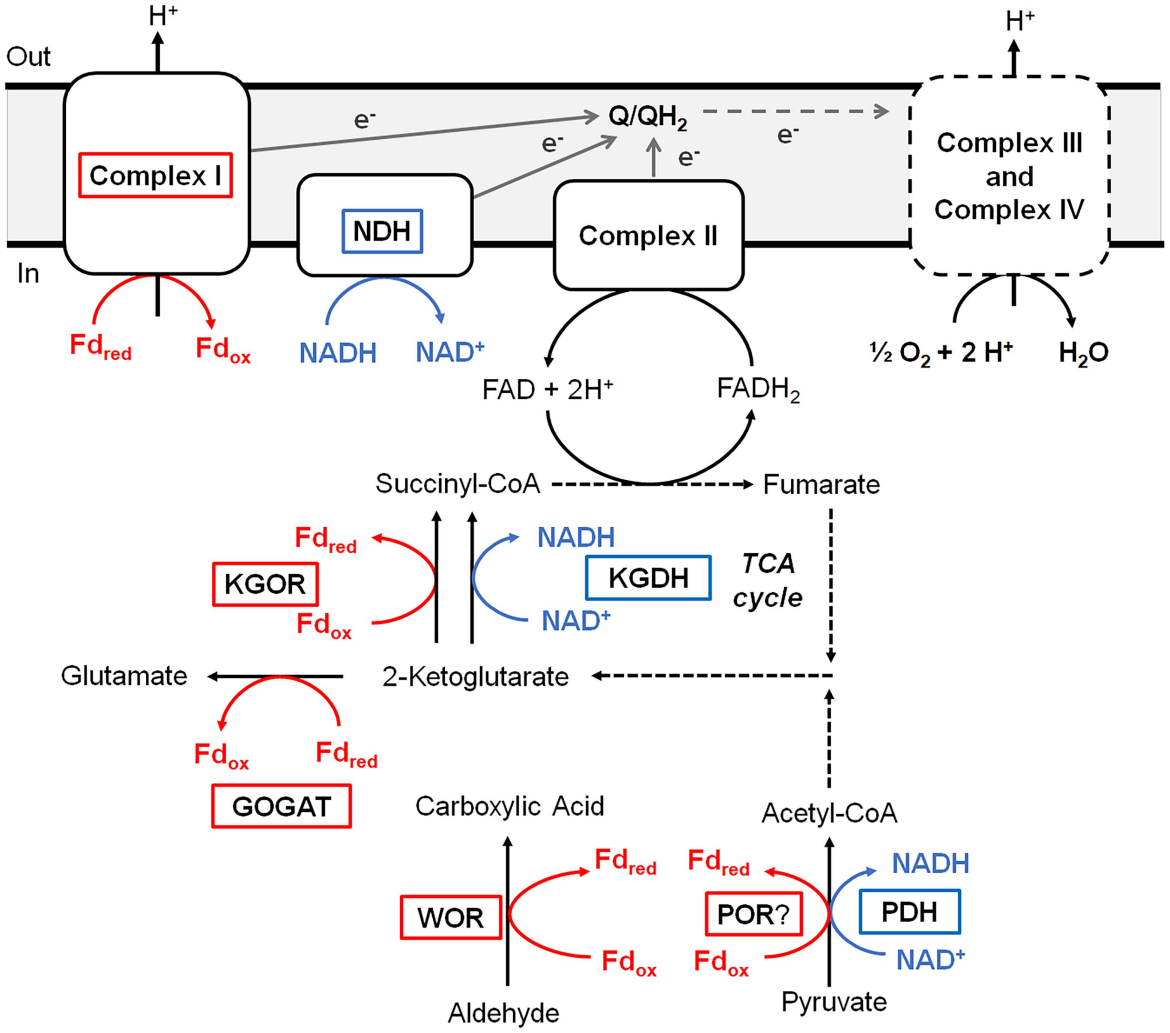
Predicted roles of WOR and other Fd-dependent enzymes during aerobic respiration of *B. massiliensis*. Aldehydes and amino acids are metabolized to CO_2_ utilizing the TCA cycle generating reduced Fd (red) and NADH (blue). Reduced Fd and NADH are then used to generate electrons that flow through the quinone pool (Q/QH_2_) before ultimately reducing O_2_ as the final electron acceptor. Fd-dependent enzymes are boxed red, NAD-dependent enzymes are boxed blue and dotted lines indicate multiple steps. See text for details.

*B. massiliensis* is an obligate aerobe and so it is very unusual for it to contain a Fd, and particularly of the 4Fe-type as these are almost exclusively found in obligately anaerobic microbes. For example, the facultative anaerobe *Escherichia coli* does not contain Fd. Moreover, as illustrated in Figure 4, Fd is the major iron-containing protein in the cytoplasm of *B. massiliensis*. The genome of *B. massiliensis* contains an operon encoding a 2-ketoacid Fd oxidoreductase (KGOR, WP_019119199.1, WP_019119200.1), as well as four encoding the expected NAD-linked 2-ketoacid dehydrogenases (WP_019119241.1, WP_019122870.1, WP_019121182.1, WP_019124010.1) and one encoding a Fd-dependent glutamate synthase (GOGAT, WP_019122315.1). This suggests that Fd could play a role in the *B. massiliensis* metabolism beyond its role as the electron acceptor for BmWOR. However, while the pyruvate Fd oxidoreductase (POR) activity in a cytoplasmic extract was extremely low (0.02 U/mg), the extracts had high KGOR activity (0.7 U/mg, using the dye-linked assay). As expected, both pyruvate dehydrogenase and 2-ketoglutarate dehydrogenase activities could also be measured but they were much lower than for KGOR (0.11 and 0.09 U/mg, respectively). This demonstrates that *B. massiliensis* uses both NAD and Fd-linked enzymes in its primary metabolism (Figure 6).

A very significant Fd-based energy metabolism in this organism is predicted from an analysis of its genome for genes encoding a respiratory Complex I or NUO system. Surprisingly, *B. massiliensis* appears to lack a conventional NADH-dependent respiratory system. Its genome encodes a Complex I/NUO that lacks the NADH input module (NuoEFG) and consists of subunits NuoABCDHIJKLMN (WP_019121088.1, WP_019121089.1, WP_019121090.1, WP_019121091.1, WP_019121092.1, WP_019121093.1, WP_007722091.1, WP_035286802.1, WP_019121095.1, WP_026074320.1, WP_019121097.1). The organism therefore contains a Fd- rather than a NADH-dependent Complex I (Schuller et al., 2019) and Fd can donate electrons directly into the aerobic respiratory chain. A single subunit non-electrogenic NADH quinone reductase is present (NDH; WP_019123757; see Figure 6) rather than a conventional NADH-dependent Complex I. However, the expected complex II, a canonical bc1 complex III, cytochrome c and three cytochrome c oxidases are all present in the genome. Hence, Fd generated by the oxidation of aldehyde and 2-ketoglutarate, as well as NADH produced by normal aerobic metabolism, can serve as electron donors for O_2_ respiration (Figure 6).

## Discussion

Our understanding of the roles W has in biology is ever expanding. The only enzyme family that almost exclusively utilizes this metal, termed WOR, was recently shown to be very diverse phylogenetically (Schut et al., 2021). It is divided into 92 distinct clades, of which only four contain a WOR with a known physiological substrate (Schut et al., 2021). Surprisingly, 24 of the WOR family clades contained representatives in organisms that are part of the UHGP catalog (Almeida et al., 2021) suggesting that W enzymes could play a role within the human gut microbiome. In fact, three WOR enzymes that were investigated, AmWOR1 and AmWOR2 from *Acetomicrobium mobile* and ElWOR1 from *Eubacterium limosum*, all oxidized aldehydes. They had broad substrate specificities and were able to detoxify multiple human gut (Wishart et al., 2018) and food (O’Brien and Morrissey, 1989) associated aldehydes to their corresponding acids. In the case of *E. limosum*, its ability to oxidize propionaldehyde was shown to be W-dependent (Schut et al., 2021).

Herein we characterized a WOR enzyme from *B. massiliensis*, which is contained in one of the 24 human gut-associated WOR clades, Clade 83. BmWOR has highest activities with aromatic aldehydes, including benzaldehyde, cinnamaldehyde and tolualdehyde. Such aromatic aldehydes have been detected in the human gut (Wishart et al., 2018) and are known to be present in various foods, including spices and pit containing fruits (O’Brien et al., 2005). BmWOR also had high furfural oxidation activity, which is present in cooked foods (O’Brien and Morrissey, 1989). Moreover, it was also shown here that the oxidation of furfural to furoic acid by *B. massiliensis* was dependent on the presence of added W to the growth medium. Assuming that BmWOR oxidizes these aromatic compounds *in vivo,* the question arises as to what *B. massiliensis* does with the corresponding acids. Its genome encodes a number of aromatic-degrading pathways, including for chlorpyrifos, paraoxon and parathion (Caspi et al., 2020) as well as two aromatic-ring-hydroxylating dioxygenase enzymes (WP_019119317.1 and WP_019121217.1). Indeed, that may explain why we did not detect benzoic acid accumulation in the spent medium from cultures grown in the presence of benzaldehyde. Interestingly, BmWOR has an extremely high affinity for all aldehydes that were tested, including non-aromatic aldehydes like isovaleraldehyde and propionaldehyde (all apparent K_m_ values < ~ 6 μM), which are also present in the gut. However, as mentioned above, both benzaldehyde and furfural are catabolized by *B. massiliensis* and we propose that there is more than sufficient W that contaminates the standard growth medium to support significant WOR activity even in the absence of added W. This conclusion is supported by the data presented in Figure 3 showing that there is measurable aldehyde oxidation activity in the cytoplasmic extract of cells grown in the absence of added W, and that this increases only about 3-fold when W is added to the growth medium. Hence, there is very significant WOR activity in the absence of added W to oxidize the exogenously added aldehydes.

Not only does BmWOR have a proposed detoxification role in a strict aerobe, it also contributes to its energy metabolism as it provides aromatic acids and reduced Fd for the aerobic aromatic degradation pathway, as discussed above. The characterized WOR family enzymes are extremely oxygen sensitive and are usually inactivated within minutes of exposure to air (Mukund and Adams, 1991, 1995; Roy et al., 1999). The AOR from *Aromatoleum aromaticum* EbN1 was previously touted as the most oxygen-tolerant purified WOR, with a half-life in air of 1 h (Arndt et al., 2019). In contrast, BmWOR retained 50% of its activity after exposure to air for 48 h. Moreover, BmWOR catalyzed the oxidation of furfural in the presence of air (~ 200 μM O_2_ in solution), which is quite remarkable given the history of this class of tungsten-containing enzyme. The fact that O_2_ is not directly reduced by BmWOR (Fd is absolutely required for aldehyde oxidation activity) may give a clue as to why this enzyme is much more oxygen-tolerant than WORs from anaerobic microorganisms. We hypothesize that the redox cofactors that are typically O_2_ reactive are protected by the BmWOR protein preventing their interaction with O_2_. Structural analysis will be required to determine if this is indeed the case.

In addition to WOR, the strict aerobe *B. massiliensis* contains other proteins typically associated with an anaerobic metabolism (Figure 6). These include the 4Fe-Fd that we purified, which is the proposed physiological electron acceptor for BmWOR, and KGOR and GOGAT enzymes. Interestingly, a [4Fe-4S]-cluster containing Fd was recently reported to be functional in the aerobic cyanobacterium *Synechocystis* sp. during photomixotrophic growth (Wang et al., 2022). This organism also contains an oxygen-resistant POR but, unlike BmWOR, this enzyme was not catalytically active in air unless glucose oxidase and catalase were also present to remove the oxygen and hydrogen peroxide from the assay (Wang et al., 2022). Based on the results presented herein, *B. massiliensis* appears to preferentially utilize Fd rather than NAD as the electron acceptor for 2-ketoglutarate oxidation in the TCA cycle. The organism also contains a Fd-dependent Complex I enzyme similar to the enzyme found in some cyanobacteria and aerobic Archaea (Schuller et al., 2019). Hence in *B. massiliensis* both aldehyde and 2-ketoglutarate oxidation are proposed to be coupled to respiratory O_2_ reduction *via* a conventional respiratory chain using an anaerobic-type 4Fe-Fd (Figure 6). Utilizing Fd-linked enzymes while growing aerobically would require an efficient scavenging system for reactive oxygen species and this seems to be the case with *B. massiliensis.* It is catalase positive (Hugon et al., 2013) and its genome encodes a catalase (WP_026074400.1), two different Mn-superoxide dismutases SOD; (WP_173391411.1 and WP_019120196.1) and a Cu/Zn-SOD (WP_019121373.1). Our metal analysis after the separation of cytoplasmic proteins on an ion-exchange column revealed many Mn and Cu peaks in the QHP column elution profile in comparable concentration ranges as the W in the WOR peak and some presumably contain the SOD enzymes (Supplementary Figure S4).

BmWOR is a robust enzyme. In addition to being aerotolerant (especially compared to other WOR enzymes), it is halotolerant (retaining 80% activity after 24 h in 3 M KCl) and thermally stable (retaining 50% activity after 20 h at 55°C). This is reflected in the Clade 83 WOR tree where related WOR enzymes are found in rather diverse organisms. These span several archaeal and bacterial Phyla, including Deinococcota, Chloroflexota, Firmicutes and Euryarchaeota. However, approximately 75% of cultured members are halophilic and require over 3% w/v NaCl (0.51 M) for growth, and many of them are aerobic and extremely halophilic (requiring up to 25% w/v or 4.3 M NaCl) Archaea. Additionally, about 22% of the cultured organisms containing a Clade 83 WOR grow optimally at temperatures greater than 45°C, including several thermophilic bacteria from the *Thermus* genus. *B. massiliensis* itself is mesophilic with a growth optimum of 37°C (Hugon et al., 2013) and can only grow at NaCl concentrations <200 mM. That BmWOR is temperature and salt tolerant beyond the growth capabilities of *B. massiliensis* may indicate that the gene encoding BmWOR gene was horizontally transferred to this organism from a phylogenetically distant species, likely within the Archaeal domain, which make up 63% of Clade 83 organisms.

Perhaps the most surprising aspect of the phylogenetic tree of Clade 83 WOR enzymes is the number that are aerobes (or facultative anaerobes) and their connections to human health and nutrition. Of the 171 WOR enzymes in cultured organisms, 73% were in aerobes (95 archaea and 29 bacteria) and of the 45 distinct genera, eight are human-related and have been found in either gut or lung tissues, and nine have been found in food-related samples, typically salt-containing and fermented, such as shrimp jeotgal and kimchi (Supplementary Table S1). In addition, 15 genera harboring a Clade 83 WOR have been found in commercial salt samples and/or salterns (Lee, 2013; Seck et al., 2018). If the properties of BmWOR are mirrored by the other Clade 83 WOR enzymes found in these aerobic, extreme halophiles, then there are interesting implications in such organisms promoting a healthy gut microbiome by the WOR-catalyzed detoxification of aldehydes. Humans are exposed to toxic aldehydes daily (Lopachin and Gavin, 2014), some of which naturally occur in foods, some are generated during the cooking process, and some are also produced endogenously in the gut microbiome (O’Brien and Morrissey, 1989; O’Brien et al., 2005). For example, propenal consumption is estimated to be 5 mg/kg/day (Conklin et al., 2010) while that of furfural is estimated to be 0.3 mg/kg/day (O’Brien et al., 2005). The high-affinity, broad-aldehyde-substrate oxidation activities of BmWOR-like enzymes could promote a healthy gut. However, such an activity could be limited in the gut by W availability as we saw BmWOR activity in *B. massiliensis* grown without added W increase ~3-fold when W was added (Figure 3). While there is little data on W concentrations in the human gut, it was reported that W concentrations in a dietary nutritional balance study were much lower than those of Mo (8 to 13 μg/day vs. 100 to 270 μg/day; Wester, 1974).

Furthermore, a systematic genomic analysis of human gut microbiota indicates that aerobic respiratory reductases are more common in human gut than previously thought, where microbes that contain them take advantage of steep oxygen gradients (Ravcheev and Thiele, 2014). Thus it would be interesting to investigate the use of organisms containing oxygen- tolerant WORs, supplemented with W, for use in animal probiotics. Spore forming organisms closely related to *B. massiliensis,* including other *Brevibacillus* and *Bacillus* strains, are currently used as commercialized probiotics (Sanders et al., 2003). Among groups of bacteria promoting health in a piglet model, *Bacillus coagulans* was shown to increase aerobic and anaerobic spore formers while decreasing fecal coliforms (Adami and Cavazzoni, 1999). BmWOR is most active in oxidizing aromatic aldehydes, particularly benzaldehyde to benzoic acid, which could also be beneficial for animal probiotics in cases where it could accumulate. Benzoic acid has been shown to have anti-fungal (Jeong et al., 2017) and antimicrobial activity (Pu et al., 2018). A probiotic combination of benzoic acid and oregano oil together with *Bacillus coagulans* was successfully used to protect piglets from enterotoxigenic intestinal injury caused by *E. coli* (Pu et al., 2018). Therefore, increased understanding of the role and scope of W metabolism in the human gut microbiome may lead to new insights both in human health and in animal probiotics.

## Data availability statement

The original contributions presented in the study are included in the article/Supplementary material; further inquiries can be directed to the corresponding author.

## Author contributions

GS, MT, FP, SP, and MA designed the study, analyzed the data, and wrote and edited the paper. GS, MT, FP, SP, HM, and WD performed the experiments. All authors contributed to the article and approved the submitted version.

## Funding

This work was supported by a grant from the National Institutes of Health (GM136885).

## Conflict of interest

The authors declare that the research was conducted in the absence of any commercial or financial relationships that could be construed as a potential conflict of interest.

## Publisher’s note

All claims expressed in this article are solely those of the authors and do not necessarily represent those of their affiliated organizations, or those of the publisher, the editors and the reviewers. Any product that may be evaluated in this article, or claim that may be made by its manufacturer, is not guaranteed or endorsed by the publisher.
