# Supplementary material for: Obligately aerobic human gut microbe expresses an oxygen resistant tungsten-containing oxidoreductase for detoxifying gut aldehydes
Source: Front Microbiol. 2022 Aug 16;13:965625. doi: 10.3389/fmicb.2022.965625 (PMC9424855; doi:10.3389/fmicb.2022.965625)
Supplement: Supplementary file 1 [file Data_Sheet_1.pdf]

Supplementary Information

Obligately Aerobic Human Gut Microbe Expresses an Oxygen Resistant Tungsten-Containing Oxidoreductase for Detoxifying Gut Aldehydes

Michael P. Thorgersen†, Gerrit J. Schut†, Farris L. Poole II, Dominik K. Haja, Saisuki Putumbaka, Harriet I. Mycroft, Willem J. de Vries and Michael W. W. Adams

Department of Biochemistry and Molecular Biology
University of Georgia, Athens, GA 30602, USA

† These authors contributed equally to this work.

Supplementary Figures 1-6
Supplementary Tables 1-2

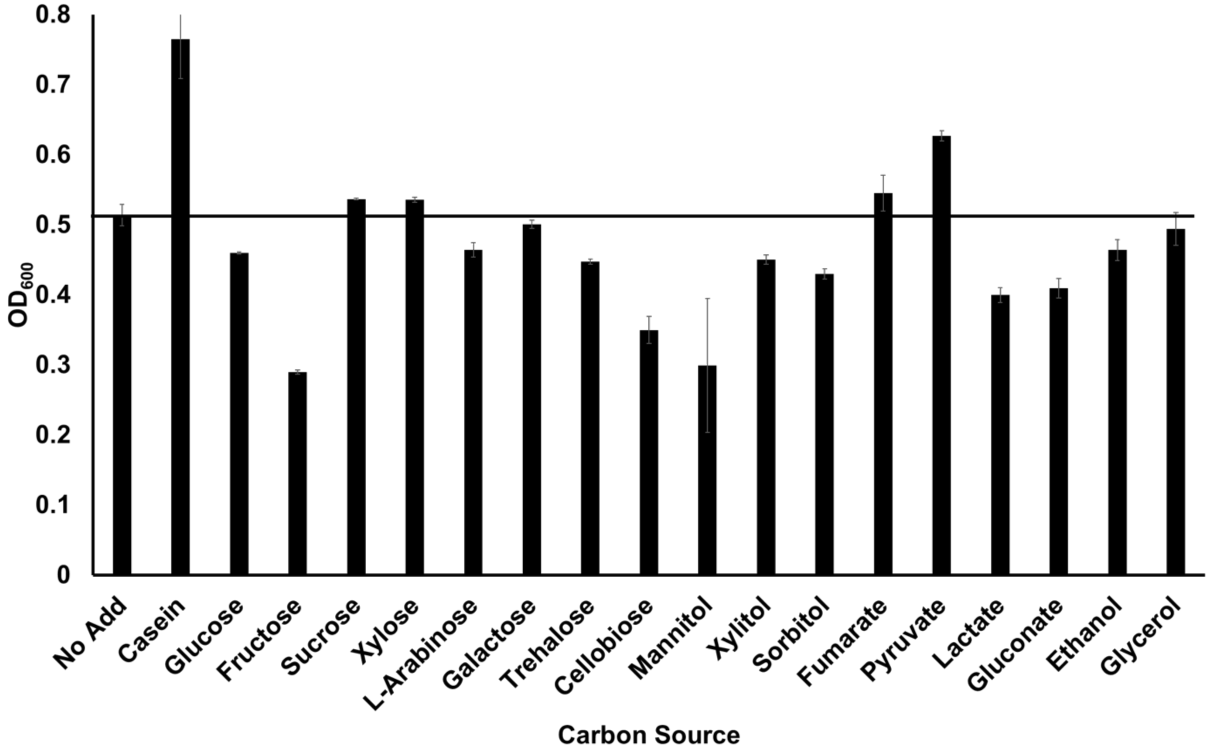

Supplementary Figure 1. Growth of *B. massiliensis* on yeast extract in the presence of other potential carbon sources. *B. massiliensis* was grown on base medium containing 0.5 g/L yeast extract with other added carbon sources, each at a concentration of 20 mM, (2.0 g/L), glycerol (5% v/v) and ethanol (0.5% v/v). Growth (OD_{600}) is reported after 60 hr and the horizontal black line indicates the growth observed with yeast extract only.

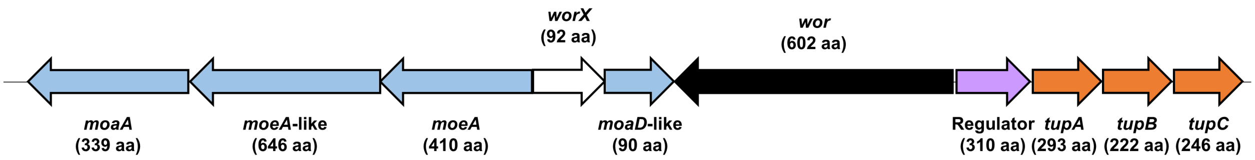

Supplementary Figure 2. Gene localization of BmWOR. The enzyme is encoded by a single gene on the opposite DNA strand to the surrounding genes, which encode a transcriptional regulator, the tungstate ABC transporter and several pyranopterin cofactor synthesis proteins, all of which are putative.

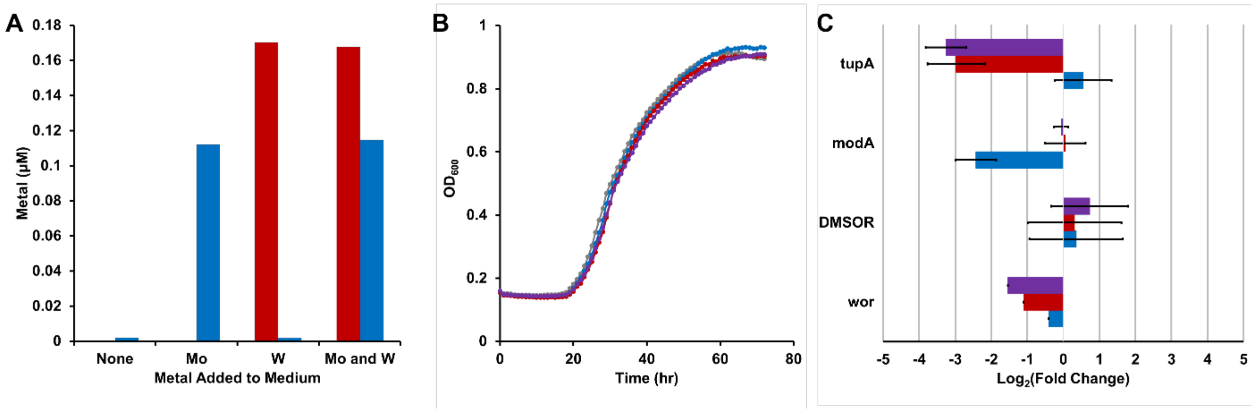

Supplementary Figure 3. Effect of W and Mo addition on *B. massiliensis* growth and expression of related genes. A) The measured concentration of Mo (blue) and W (red) in the growth medium with and without addition of W and/or Mo (100 nM) (performed in singlicate). **B)** Growth of *B. massiliensis* with no additions (grey), added W (red, 100 nM), added Mo (blue, 100 nM) and added W and Mo (purple, each 100 nM). **C)** QPCR was used to determine the change in expression ($\log_2$ fold change) of W and Mo related genes between cells grown as described in B) with added W (red), added Mo (blue) and both added (purple) relative to no metal additions. Expression levels are relative to the GAPDH1 gene (WP_019121966.1).

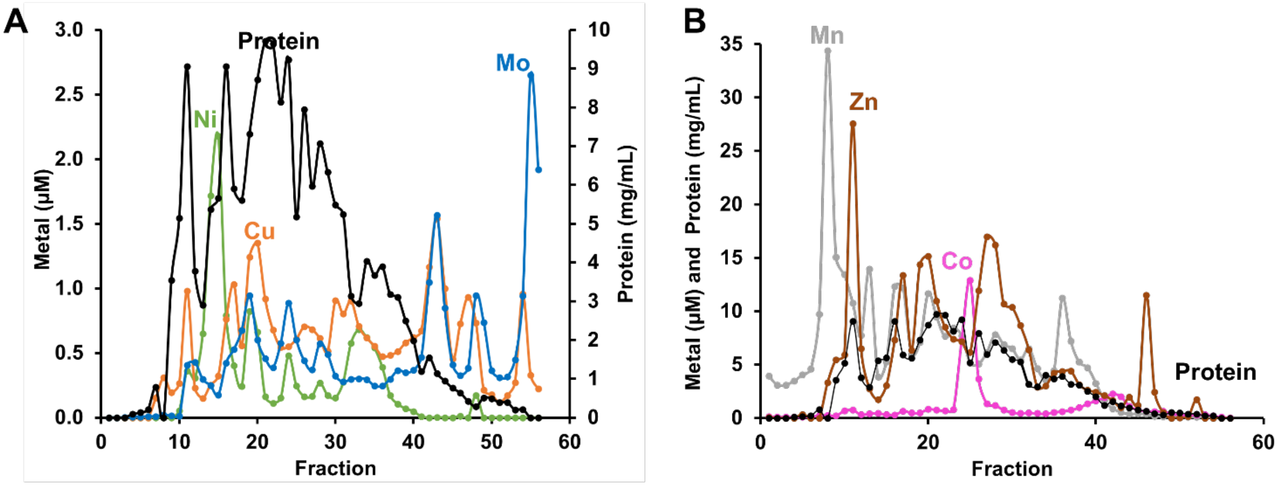

Supplementary Figure 4. Concentrations of various metals in the QHP column fractions. The cytoplasmic extract of *B. massiliensis* cells (50 g, wet weight) was fractionated using an anion exchange QHP column and the fractions were analyzed for **A)** protein (black), Ni (green), Cu (orange) and Mo (blue), and **B)** Mn (grey), Co (Pink), Zn (brown).

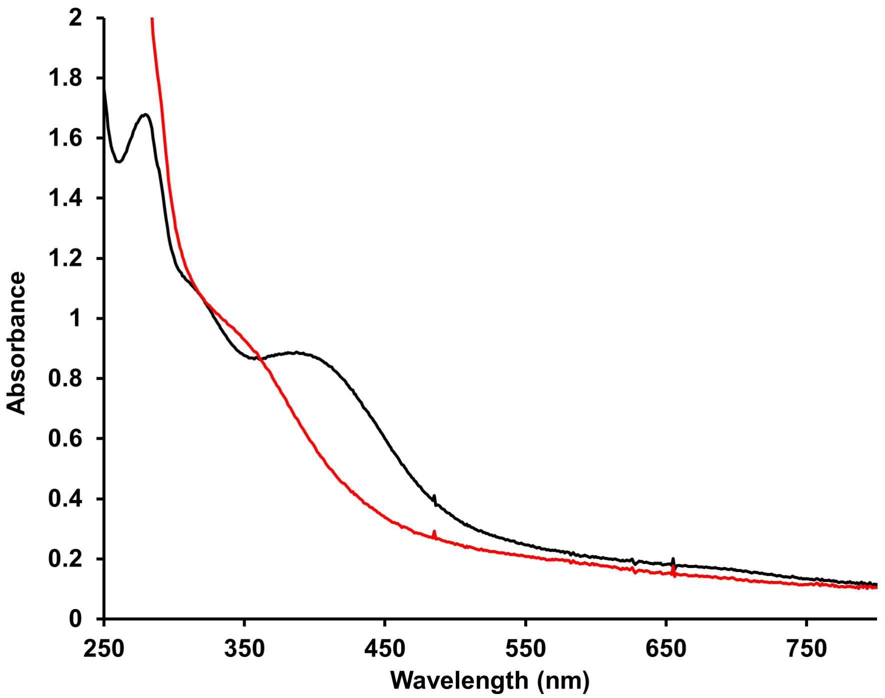

Supplementary Figure 5. UV-visible spectra of *B. massiliensis* ferredoxin. Oxidized *B. massiliensis* Fd (50 μM, black line) was reduced with an excess of Ti(III) citrate (red line).

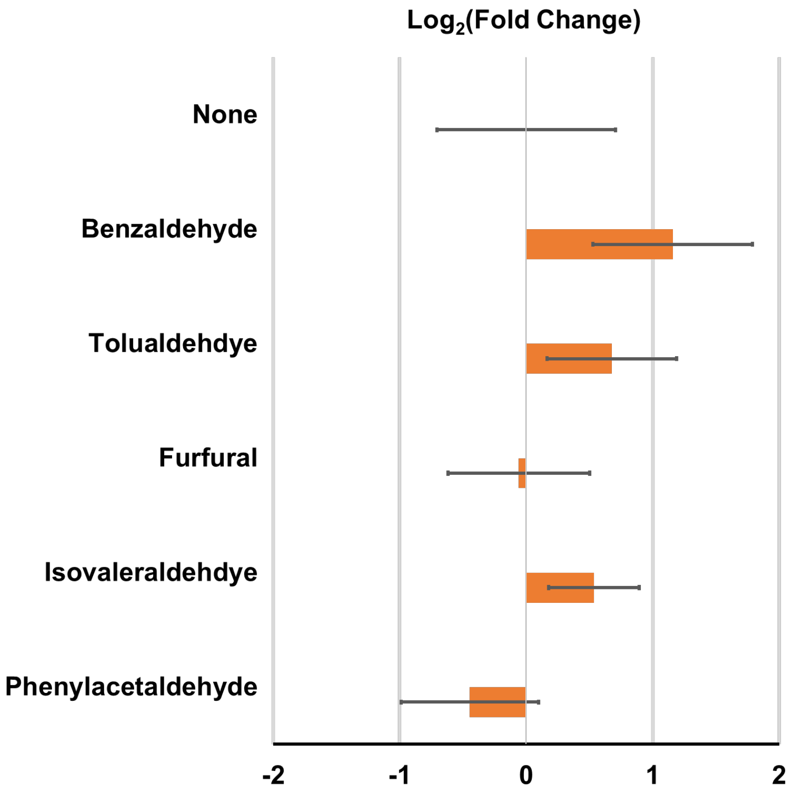

Supplementary Figure 6. Expression of the *wor* gene in *B. massiliensis* cells grown in the presence of different aldehydes. Aldehydes were at a concentration of 1 mM. The expression level of *wor* is relative to the GAPDH1 gene (WP_019121966.1) given as the $\log_2$ value.

Supplementary Table 1. Properties of the 197 microorganisms that contain a Clade 83 WOR. Those that are cultured and were identified to be human-, food- or salt-associated are designated with a Y in the respective column. Cultured microorganisms are classified by temperature (mesophile, thermophile or hyperthermophile), salt requirement (slight, moderate or extreme halophile and/or halotolerant), pH (alkaliphile or neutrophile) and oxygen (anaerobe, aerobe or facultative anaerobe). These classifications are used in **Figure 1** and the references are listed. Several microorganisms that have unique Clade 83 WOR sequences but have identical species names because they are non-cultured, have multiple WORs or are poorly named strains. See the Materials and Methods section for classification criteria references.

(supplied as an Excel file)

Table S2. Oxidation of aldehydes by *B. massiliensis* cytoplasmic extract and purified BmWOR. Activities were measured anaerobically at 25°C in 96-well plates using 0.25 mM of the indicated aldehyde as the substrate and 1 mM benzyl viologen as the electron acceptor. The results are expressed as substrates having >50% activity of the activity with benzaldehyde (++), having detectable activity <50% of the activity with benzaldehyde (+), and having no detectable activity (-). The cytoplasmic extract and purified BmWOR had 0.3 U/mg and 7.1 U/mg activity with benzaldehyde respectively. Aldehydes highlighted in blue have been detected in the human gut metabolome while those in green correspond to acids found in the human gut metabolome and those in yellow are found in cooked foods (see text for details).

Aldeheyde	Cell Free Extract	BmWOR
Tolualdehyde	++	++
Benzaldehyde	++	++
Cinnamaldehyde (trans)	++	++
Glycoaldehyde	++	++
Furfural (2-furaldehyde)	++	++
3,4-Dihydroxybenzaldehyde	++	++
2-Methyl-2-butenal	++	++
2-Hydroxylbenzaldehyde	+	+
5-Hydroxymethylfurfural	+	+
2-Ethylisovaleraldehyde	+	+
4-Hydroxybenzaldehyde	+	+
Vanillin	++	+
Phenylacetaldehyde	++	+
3-Hydroxybutanal	+	+
4-Hydroxy-3-methoxycinnamaldehyde	++	+
Formaldehyde	+	+
o-Aminobenzaldehyde	+	+
Trans-2-octenal	+	+
Pentanal	-	+
Syringaldehyde	-	+
Salicaldehyde	-	+
2-Ethylbutyraldehyde	-	+
Indole-3-carbaldehyde	-	+
3,5-Dimethoxy-4-hydroxycinnamaldehyde	-	+
2-Methylvaleraldehyde	-	+
Glutaraldehyde	-	+
Propionaldehyde	-	+
2,5-Dimethoxybenzaldehyde	-	-
2-Ethylhexanal	-	-
2-Methoxybenzaldehyde	-	-
2-Methylbutyraldehyde	-	-
2-Phenylpropionaldehyde	-	-
Acetaldehyde	-	-
Butyraldehyde	-	-
Crotonaldehyde	-	-
Decanal	-	-
Dodecyaldehyde	-	-
Glyceraldehyde	-	-
Glyoxal	-	-
Hexanal	-	-
Isobutyraldehyde	-	-
Isovaleraldehyde	-	-
Methyl glyoxal (pyruvic aldehyde)	-	-
Nonanal	-	-
Octanal	-	-
Phenylpropionaldehyde	-	-
Phthaldialdehyde	-	-
Terephthaldehyde	-	-
